# Variation in the Bandgap of Amorphous Zinc Tin Oxide: Investigating the Thickness Dependence *via In Situ* STS

**DOI:** 10.1021/acsomega.3c09958

**Published:** 2024-01-31

**Authors:** Peter J. Callaghan, David Caffrey, Kuanysh Zhussupbekov, Samuel Berman, Ainur Zhussupbekova, Christopher M. Smith, Igor V. Shvets

**Affiliations:** † School of Physics and Centre for Research on Adaptive Nanostructures and Nanodevices (CRANN), Trinity College Dublin, Dublin 2, Ireland; ‡ School of Chemistry, Trinity College Dublin, Dublin 2, Ireland; § L.N. Gumilyov Eurasian National University, 2 Satpayev Street, Astana 010000, Kazakhstan

## Abstract

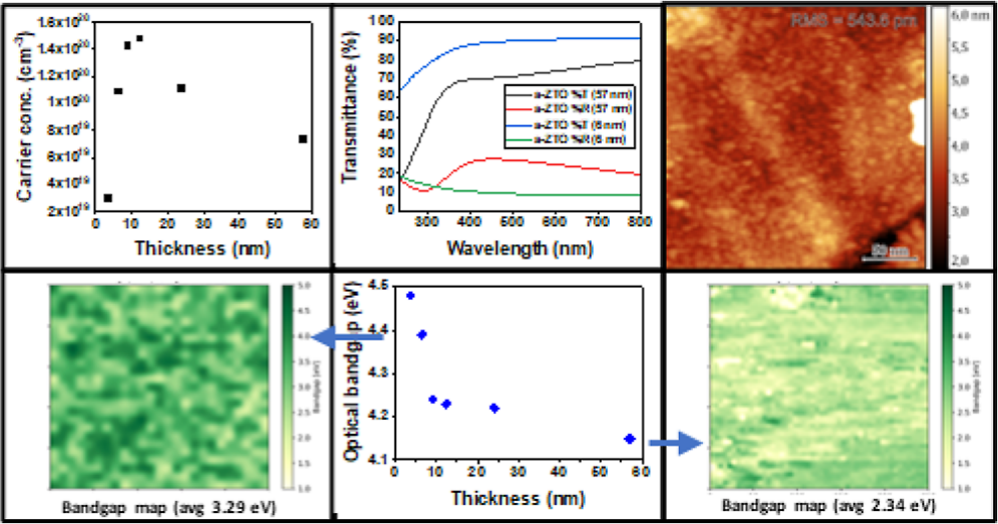

Amorphous transparent conducting oxides (a-TCOs) have seen substantial interest in recent years due to the significant benefits that they can bring to transparent electronic devices. One such material of promise is amorphous Zn_*x*_Sn_1–*x*_O_*y*_ (a-ZTO). a-ZTO possesses many attractive properties for a TCO such as high transparency in the visible range, tunable charge carrier concentration, electron mobility, and only being composed of common and abundant elements. In this work, we employ a combination of UV–vis spectrophotometry, X-ray photoemission spectroscopy, and *in situ* scanning tunneling spectroscopy to investigate a 0.33 eV blue shift in the optical bandgap of a-ZTO, which we conclude to be due to quantum confinement effects.

## 1 Introduction

Amorphous transparent conducting oxides (a-TCOs) are an alternative to crystalline TCOs that have garnered interest in recent years.^1−5^ In a-TCOs, the conduction band minimum (CBM) is typically composed of the *n*s orbital, where *n* is the primary quantum number. If the radius of the s-orbital is greater than the intercation distance, as happens for *n* > 4, then strong conduction pathways can exist even in the absence of long-range order in the material.^6,7^ a-TCO materials such as indium–zinc-oxide grown at room temperature have shown conductivity values comparable to those of crystalline Sn-doped In_2_O_3_ (ITO).^8^ This room-temperature growth is advantageous because it is below the glass temperature of the plastic substrates often used in flexible electronics, such as polyethylene terephthalate. In comparison, polycrystalline ITO thin films often require deposition temperatures >400 °C,^9^ far exceeding the temperature budget of plastic substrates.^10^ One challenge that many a-TCO materials face, however, is their reliance on In and Ga to achieve high conductivities. In addition to the arguments regarding their scarcity of supply,^11^ there are numerous health concerns surrounding the use of both elements.^12−14^

In this study, we examine an alternative a-TCO, amorphous Zn_*x*_Sn_1–*x*_O_*y*_ (a-ZTO). Both ZnO and SnO_2_ are well-known n-type TCOs and have been shown by Hautier *et al.* to possess among the highest electron mobilities among all binary oxides.^15^ The arguments for a-ZTO are numerous: a-ZTO is only composed of common and abundant elements;^16^ it has been shown to possess high mobilities even in an amorphous state;^17^ and it has already been implemented in a wide range of applications such as organic light-emitting diodes (O-LEDs),^18^ photovoltaic cells,^19−21^ and thin film transistors (TFTs).^22,23^ It is imperative to understand how key properties such as morphology, bandgap, and conductivity are affected in the low-thickness regime as these properties often play a decisive role when it comes to selecting the compatibility of a material in a device.^24^

When one considers the wide bandgap of zinc tin-oxide (ZTO), investigating its morphological, optical, and electronic properties can be complicated, especially in the case of the amorphous state. The core peak and valence band structure of a-ZTO have been reported in photoemission spectroscopy (PES) studies,^16,25,26^ but properties such as conductivity and bandgap predominately rely on the position and structure of the conduction band (CB). PES, however, cannot probe the CB, and angle-resolved photoemission spectroscopy is not appropriate for a-ZTO. Electron energy loss spectroscopy (EELS) can be applied to measure the bandgap of a-ZTO; however, without a monochromated electron source, the energy resolution is often below that of the more accessible UV–vis spectroscopy. An appropriate method to resolve both the valence band (VB) and CB edges is scanning tunneling spectroscopy (STS).^27^ STS offers localized and nondestructive measurements that can therefore be utilized to define the electronic bandgap of a material. The localized measurement capability of STS means that it can probe the electronic structure of materials at the nanoscale, allowing for a detailed understanding of inhomogeneities and local variations in electronic properties that might be missed by bulk measurement techniques. To the authors’ knowledge, STS measurements have not yet been applied to probe the electronic bandgap of a-ZTO films.

In this paper, we examine the thickness dependence of the bandgap in a-ZTO thin films using both UV–vis spectrophotometry and STS. We demonstrate that at lower thicknesses, a blue shift in bandgap occurs. To conclude, we propose some possible origins for this effect.

## 2 Methodology

a-ZTO thin films were grown *via* nonreactive RF magnetron sputtering in an Ar atmosphere. The base pressure in the chamber was 1 × 10^–5^ mbar. The substrate was a standard microscope glass slide for basic electrical or thickness measurements and quartz for UV–vis measurements. Samples were grown from a single custom a-ZTO target with a ratio of 30% Zn to 70% Sn. Prior to deposition, a target presputter was performed for 10 min to clean the surface of the sputtering target. Samples were grown at 300 °C in a pure Ar atmosphere at a pressure of 1 × 10^–3^ mbar.

The conductivity, Hall mobility, and charge carrier concentrations of the samples were measured using a four-point probe arrangement and an 800 mT electromagnet. Electrical connections in the van der Pauw configuration were made by using silver wires that were adhered to each corner of the sample with silver paint.

UV–visible spectrophotometry was performed with the *PerkinElmer Lambda 650S* and was used to measure the transmission and reflectance of the films in the range (1.5–5) eV. The absorption coefficient (α) was calculated from the transmission and reflectance data using eq 1.
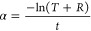
1where *T* and *R* are the transmission and reflectance of the films, respectively, at a given energy, and *t* is the thickness of the films. Tauc plots of (α*h*ν)^1/γ^*vs h*ν, where *h* is Planck’s constant, ν is the photon frequency, and γ is a constant that depends on the nature of the transition, were performed in order to determine the optical band gap.

A commercial *Createc* low-temperature scanning tunneling microscope was utilized to examine the a-ZTO thin films. A vacuum suitcase with a base pressure of 2 × 10^–10^ mbar was used to transfer a-ZTO films between the deposition chamber and the scanning tunneling microscopy (STM) system under UHV conditions, and this allowed a pristine surface to be imaged.^28^ Due to the comprehensive nature of the measurement, STS analysis was conducted on only two samples, which were selected for their extreme thicknesses. Gold contacts were deposited on the four corners of the substrate of dimensions 1 × 1 cm^2^ to improve electrical contact with the sample. The STM and STS measurements were conducted in UHV with a base pressure of 3 × 10^–11^ mbar. STM images and STS spectra were recorded at a temperature of 77 K in constant current mode. Single-crystalline W-tips with a (001) orientation were used, which were electrochemically etched in NaOH solution. The tunneling bias was applied to the sample.

Scanning electron microscopy (SEM) images were taken by using a *Zeiss Ultra* scanning electron microscope at an operating voltage of 5 kV. X-ray diffraction (XRD) measurements were performed with a *Bruker D8 Advance* X-ray diffractometer using an unmonochromated Cu K_α_ source (1.54 Å) at 40 kV and 40 mA through a 0.1 mm slit at an angle of (10–70) °. The thicknesses of samples were calculated using X-ray reflectometry (XRR) measurements with a *Bruker D8 Discover* High Resolution XRD instrument with a monochromated Cu K_α_ X-ray source (1.54184 Å). X-ray photoemission spectroscopy (XPS) was utilized to examine the core-level peaks of a-ZTO using an *Omicron MultiProbe* XPS system equipped with a monochromatic Al Kα source (XM 1000, 1486.7 eV). The instrument has a base pressure of 5 × 10^–11^ mbar and an instrumental resolution of 0.6 eV.

## 3 Results and Discussion

**Figure 1:**
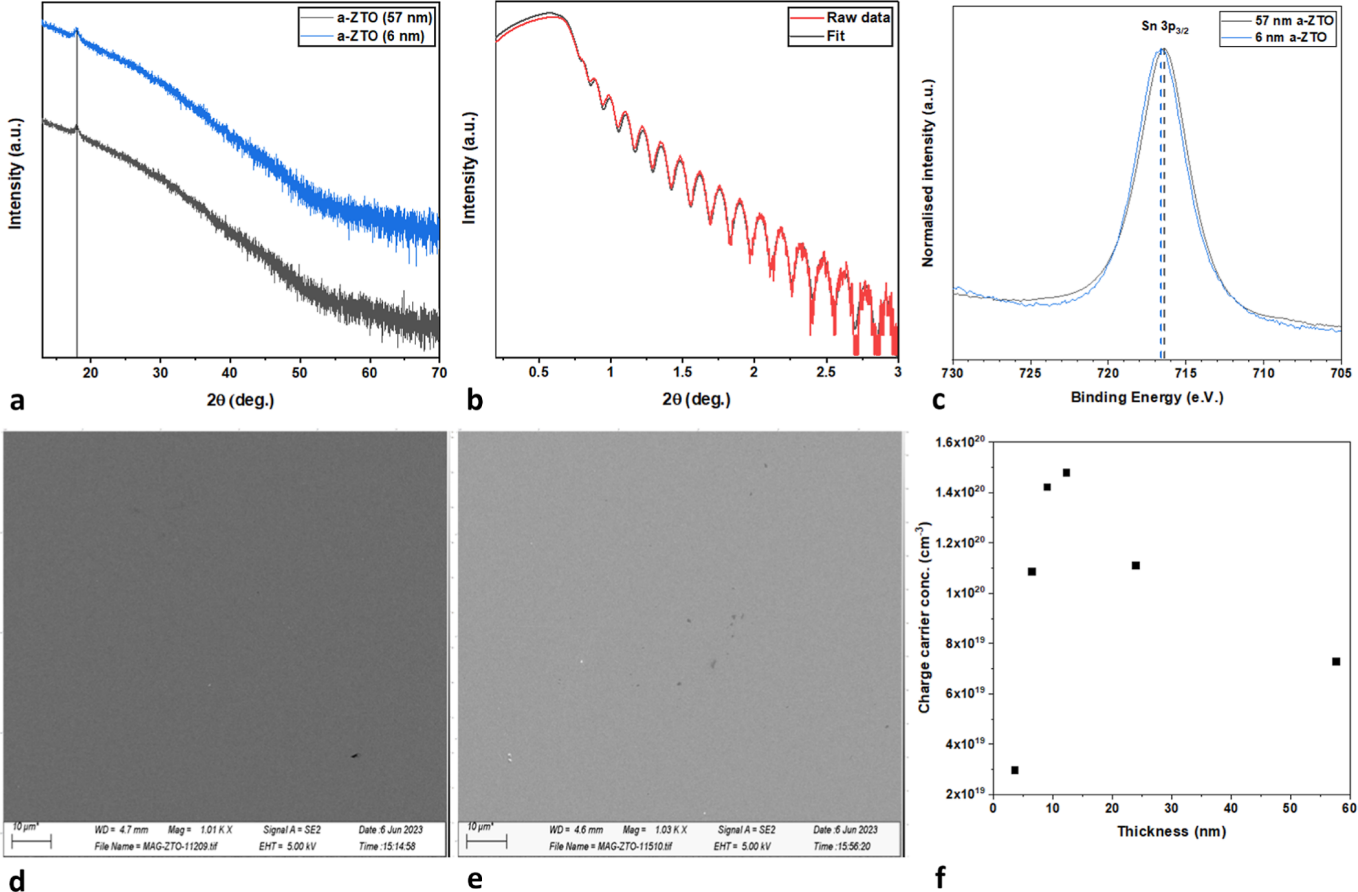
(a) XRD spectra of 57 and 6 nm a-ZTO. (b) XRR spectra from a 57 nm film and the corresponding fit. (c) Normalized XPS spectra of the Sn 3p_3/2_ core level peaks in both 57 and 6 nm samples. SEM imaging of (d) high-thickness (57 nm) and (e) low-thickness (6 nm) films. (f) Change in the carrier concentration of the films as a function of thickness.

High-quality a-ZTO thin films ranging in thickness from 3–57 nm were produced by nonreactive RF magnetron sputtering. Without postgrowth treatment, these films showed excellent candidate properties for a TCO material with below 1% absorption in the visible range, along with high charge carrier concentrations resulting in a peak conductivity of 394 S/cm. The amorphous nature of the films was confirmed by XRD measurements in Figure 1a, with only a small peak from the quartz substrate being observed at 18°, while SEM imaging of the thin films (see Figure 1d,e) indicates a highly uniform continuous surface without granular structure. XRR was employed to examine the thickness of the films (Figure 1b). The change in the carrier concentration of the films as a function of thickness is shown in Figure 1f. The elemental composition of the thin films was examined through an *in situ* XPS study. The Zn/Sn ratio in the sputtering target (30/70 = 0.43) resulted in a measured Zn/Sn ratio of 0.25 in the films. Figure 1c shows a comparison between the Sn 3p_3/2_ core level peak in 57 and 6 nm a-ZTO thin films. The fixed peak positions and peak shapes suggest the chemical composition remains consistent across films of differing thicknesses. This is consistent with the Zn 2p_3/2_ core level peak (see Supporting Information).

In order to estimate the optical bandgap of these a-ZTO thin films, the energy-dependent absorption coefficient (α) was determined experimentally from transmittance and reflectance measurements. The Tauc equation states that the optical bandgap of an amorphous semiconductor (*E*_g_) can be expressed in terms of α, as shown in eq 2.^29,30^
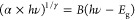
2where *h* is Planck’s constant, ν is the photon frequency, γ is a constant that depends on the nature of the transition, and B is a constant.

**Figure 2:**
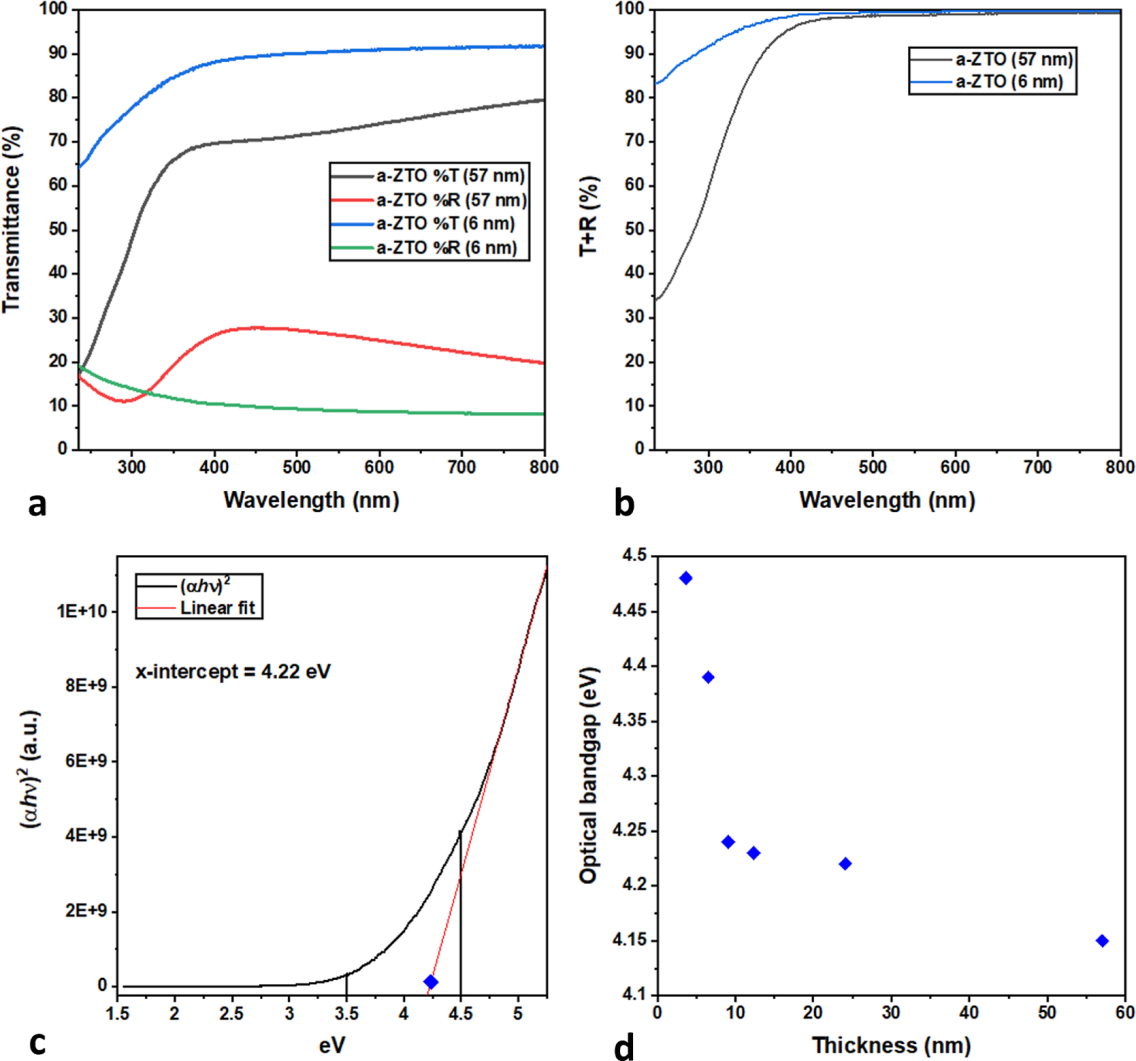
(a) Overlying transmission and reflectance spectra and (b) combined transmission and reflectance spectra for high-thickness (57 nm) and low-thickness (6 nm) a-ZTO samples. (c) Tauc diagram of a 24 nm thick film; due to the high density of subgap states, the point of linearity becomes blurred, see 3.5–4.5 eV. (d) A blue shift in the optical bandgap of a-ZTO thin films is observed as thickness is reduced.

Before discussing the results of the Tauc plot, it is worth highlighting the limitations of such plots in cases of high subgap density of states (DOS), a prominent feature of amorphous TCOs. A high density of subgap states is a well-known consequence of the disorder of amorphous materials. Such subgap states can introduce errors into Tauc diagrams by blurring the point at which the absorption edge becomes linear. This occurs as the subgap DOS creates a low slope increase region, see 3.5–4.5 eV in Figure 2c. In this work, care is taken to ensure that measurements are obtained from the linear region at higher energies to avoid this broad onset. In addition, there can also be the introduction of a “turn-down” region as error is introduced by the transmission spectra saturating at higher energy. By working with films of sub-100 nm thickness, the films in use see significant (10–20%) transmission even at higher energy, see Figure 2a, thus avoiding these issues in this work.

Figure 2d shows the calculated bandgaps of a number of a-ZTO samples as the thickness is varied in the range of 3–57 nm. A clear blue shift, the increase in value of the optical band gap (*E*_g_) with a reduction in thickness of the a-ZTO thin films, is observed. Some mechanisms that may be responsible for this 0.33 eV shift are discussed herein.

The Moss–Burstein shift is a common occurrence in TCO materials. Charge carriers will preferentially populate the lowest available energy state in the CB. As the charge carrier density increases, new charge carriers must be excited to higher energies, leading to an effective widening of the optical bandgap.^31,32^ The observed behavior, however, indicates that the charge carrier concentration decreases as the thickness is reduced (see Figure 1f); thus, carrier density-related Moss–Burstein shifts can be ruled out as the cause of this blue shift. A more likely cause is quantum confinement. Quantum confinement is an effect that occurs when charge carriers (electrons or electron holes) are confined in one or more dimensions. Electrons typically behave as free particles when the dimensions of the defining structure are very large compared to the *de Broglie wavelength*. At this scale, electron energy levels can be treated as continuous bands. When the dimensionality of the system is reduced such as in thin films (2D), nanowires (1D), or quantum dots (0D), these energy levels cease to act as continuous bands and instead become discrete.^33^ This change in band structure has knock-on implications for key properties such as optical bandgap,^34−36^ conduction state (semiconductor, semimetal, etc.),^37,38^ and photoluminescence.^39^ In 2D transition metal dichalcogenides such as PtSe_2_, a rise in bandgap due to quantum confinement is commonly observed.^37,40^ Quantum confinement effects have also been predicted computationally for crystalline oxide systems such as SrTiO_3_, BaTiO_3_, and KTaO_3_.^35,36^ Recently, quantum confinement resulting in a blue shift in optical data has also been observed experimentally in polycrystalline TCOs such as In_2_O_3_^41^ and ZnO.^42,43^

Labram et al. describe that a thin film of thickness L can be thought of as an infinite quantum well (IQW). The energy of conduction band states available to electrons confined to an infinite quantum well can be described by eq 3
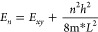
3where *E*_*xy*_ is the energy associated with the electron in the unconfined *xy*-plane, *n* is a positive integer, *h* is the Planck constant, m* is the effective mass of electrons in the semiconductor, and *L* is the thickness of the quantum well in the *z*-direction where the *z*-direction is defined as parallel to the surface normal.^43^ One can clearly see that as the thickness of the films *L* decreases, the energy of the CBM (*n* = 1) increases, resulting in a blue shift in the energy required to excite an electron to the conduction band.

In addition to the XPS core level spectra shown in Figure 1c, high-resolution XPS scans of the valence band maximum (VBM) were recorded (Supporting Information). These showed no significant change in the VBM structure for films of varying thickness values. This implies that the change is primarily located in the CB; this behavior is in agreement with the IQW model suggested by Labram et al.^43^

**Figure 3:**
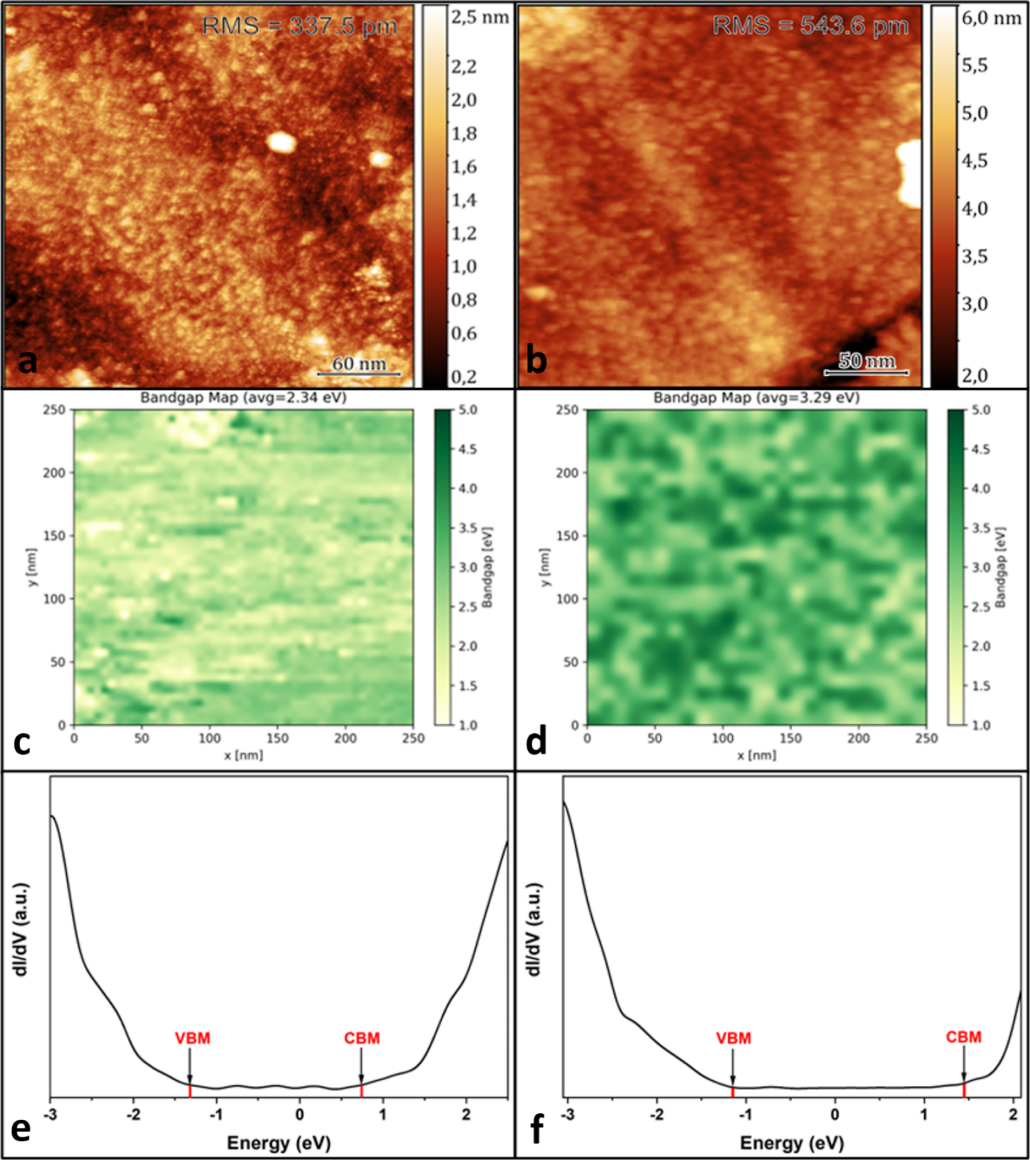
STM images of a 6 nm thin film with a (a) 300 nm × 300 nm area and a (b) 250 nm × 250 nm area show a smooth and continuous surface. (c,d) show interpolated STS grid spectra of 57 nm and 6 nm films, respectively. (e,f) area avegared dI/dV curves for 57 nm and 6 nm films, respectively; red drop lines have been added as a visual guide for bandgap estimation.

While Tauc is a strong methodology for determining the bandgap, it contains within it a number of assumptions about the nature of the bandgap. Thus, in order to support the idea that the changes being observed are real and not just an aspect of the fitting methodology, the bandgap was also measured by alternative means. As mentioned previously, STS has the ability to nondestructively probe both the VB and CB edges with very fine precision, allowing one to punctiliously define the electronic bandgap of a material. STS therefore makes for an attractive methodology to further investigate this bandgap blue shift. A 6 nm film and a 57 nm film were grown as described above and transferred *in vacuo* to the STM. STM images of the 6 nm sample, as shown in Figure 3a,b, show a continuous surface void of any preferential growth directions or anti-islands; this is in agreement with SEM analysis (see Figure 1d,e). Through STS, the electronic bandgap at a single point of the thin film could be measured, see Figure 3e,f. These spectra were built up to create a series of heat maps of the bandgap in the thin films. A blue shift is observed between the average electronic bandgap of the 57 nm film (2.56 ± 0.27) eV and the 6 nm film (3.04 ± 0.25) eV. This is due to a discernible shift in the position of the CB in the 6 nm sample.

It is noted that both samples show a surface with a large dispersion of bandgap values. Therefore, in order to portray a more accurate representation of the bandgap variation across the sample, interpolated bandgap heat maps for the 57 and 6 nm films are shown in Figure 3c,d, respectively.

This dispersion in bandgap values is due, in part, to the extremely localized nature of STS measurements (usually subnanometer) *vs* the length scale of the pixel in the grid scan (6 nm). The grid pixel may represent an area at least an order of magnitude larger than the area of measurement, resulting in this ostensibly large variation in bandgap value from pixel to pixel. Another factor is the amorphous nature of the sample in question. The composition of the samples is not perfectly homogeneous, resulting in small clusters of ZnO and SnO_2_ as well as higher-order O polymorphs.^4,44^ Further STS bandgap heat maps are available in the Supporting Information.

One must be cautious in comparing the exact bandgap figures from UV–vis measurements and STS as they are based on entirely different methodologies. In STS, one measures the highest lying occupied states in the VB and the lowest lying unoccupied states in the CB. In an amorphous thin film, it is extremely likely that these are tail states. However, when estimating the bandgap from UV–vis spectrophotometry and the Tauc method, it is these tail states that one takes care to avoid. With these considerations taken into account, one can state that STS agrees with the observation of a blue shift in the bandgap for a reduction of film thickness in a-ZTO.

To further investigate and confirm the cause of this behavior, detailed calculations would be required on the effect of confining a-ZTO or a focused examination of the CB structure using techniques such as inverse photoemission spectroscopy, X-ray absorption spectroscopy, or high-energy resolution EELS. Both of these approaches are beyond the scope of this current work, but each could play a vital role in determining the root mechanism responsible for this bandgap shift in a-ZTO.

## 4 Conclusions

To summarize, an increase of bandgap with reduced thickness is reported for a-ZTO. Below 10 nm, a significant blue shift (0.33 eV) in the optical bandgap is observed. The presence and scale of the shift were confirmed *via* STS measurements (0.48 ± 0.26) eV. While the two techniques are based on differing principles and therefore do not measure the same total gap, the shift in both cases is found to be consistent. Core peak and valence band XPS spectroscopy showed no difference between the 6 and 57 nm samples, pointing to the shift occurring in the CB. The authors note that this would be consistent with quantum confinement effects in line with the IQW model outlined above. This work demonstrates that the bandgap of a-ZTO can be controlled *via* modification of the material thickness and opens the door for a more substantial investigation of the underlying cause.

## Supporting Information Available

XPS spectra of the Zn 2p_3/2_ peak and VBM and STS heatmaps of the bandgap in the films (PDF)
