# Supplementary material for: Variation in the Bandgap of Amorphous Zinc Tin Oxide: Investigating the Thickness Dependence via In Situ STS
Source: ACS Omega. 2024 Jan 31;9(6):7262–8. doi: 10.1021/acsomega.3c09958 (PMC10870296; doi:10.1021/acsomega.3c09958)
Supplement: Supplementary file 1 — ao3c09958_si_001.pdf [file ao3c09958_si_001.pdf]

Variation in Bandgap of Amorphous Zinc Tin Oxide: Investigating the thickness dependence via in-situ STS

Peter J. Callaghan,*,† David Caffrey,† Kuanysh Zhussupbekov,‡ Samuel Berman,† Ainur Zhussupbekova,*,‡ Christopher M. Smith,† and Igor V. Shvets†

†*School of Physics and Centre for Research on Adaptive Nanostructures and Nanodevices (CRANN), Trinity College Dublin, Dublin 2, Ireland*

‡*School of Chemistry, Trinity College Dublin, Dublin 2, Ireland*

¶*L.N. Gumilyov Eurasian National University, 2 Satpayev Street, Astana, 010000 Kazakhstan*

E-mail: pcallagh@tcd.ie; zhussupa@tcd.ie

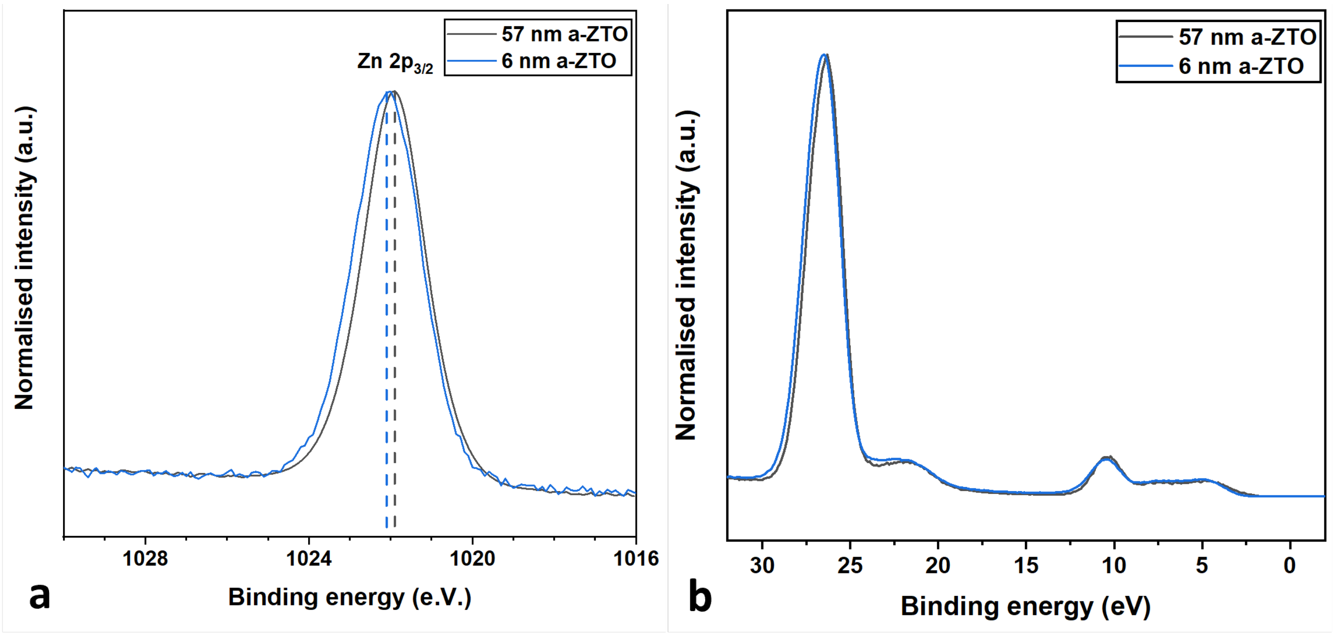

Figure S1: a) A normalised core level scan of the Zn $2p_{3/2}$ shows comparable cation core level peaks in both a 57 nm and 6 nm sample. A small peak shift is seen between the two peak positions but is below the energy resolution of the XPS instrument. b) Normalised XPS spectra of the valence band maximum region, the uniformity in the spectra suggests it is not a change in VBM structure that is causing the bandgap blueshift

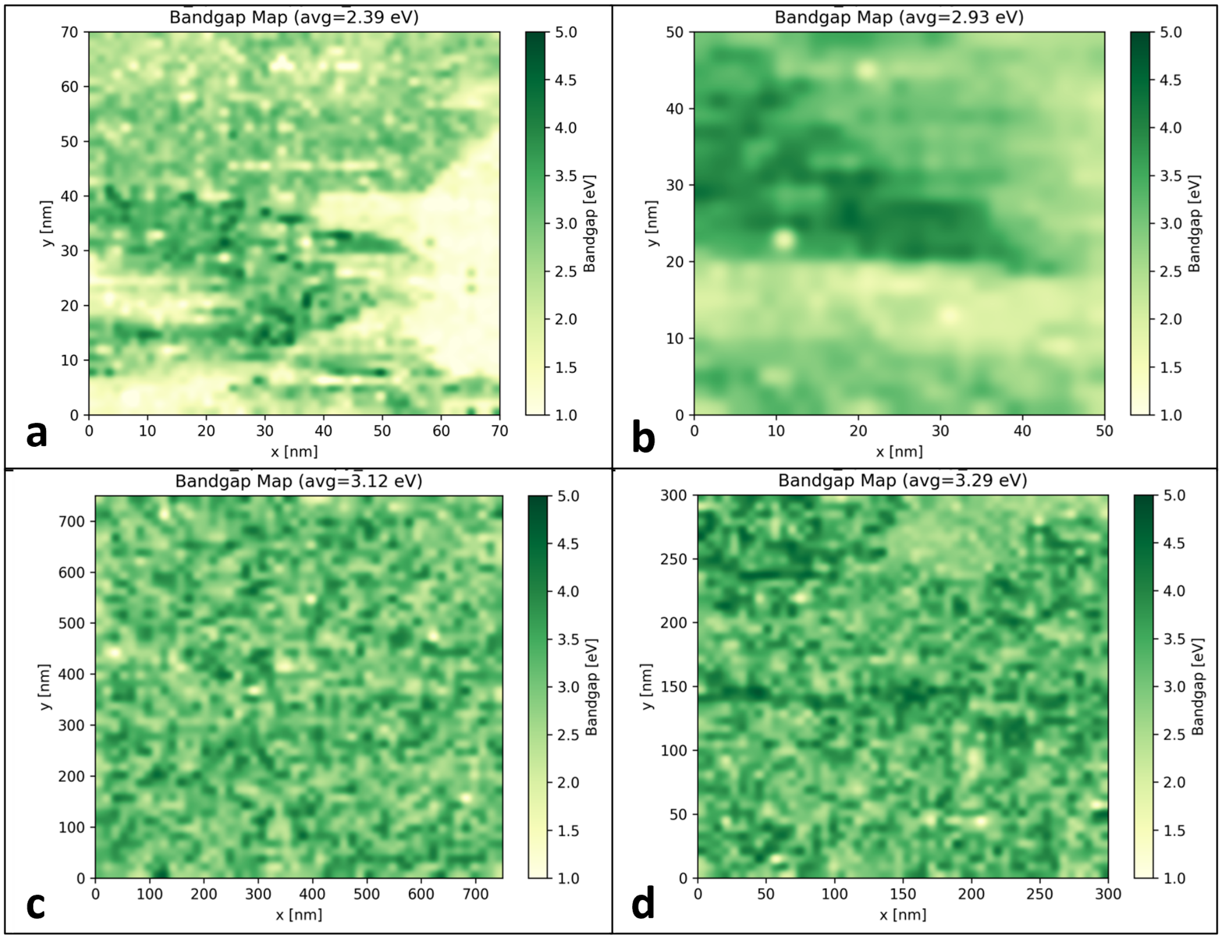

Figure S2: a) and b) a selection of interpolated STS heatmaps from a 57 nm a-ZTO thin film and a 6 nm sample c) and d).
